# Chemical Analyses and Therapeutic Properties of Plant Extracts

**DOI:** 10.3390/molecules30030610

**Published:** 2025-01-30

**Authors:** Elizabeth I. Opara, Kenneth N. White, Peter Orji Uvere

**Affiliations:** 1 School of Human Sciences, London Metropolitan University, London N7 8DB, UK; kenneth.white@londonmet.ac.uk; 2 Department of Food Science and Technology, University of Nigeria, Nsukka 410105, Nigeria; peter.uvere@unn.edu.ng

## 1. Introduction

It has been almost 20 years since the World Health Organization (WHO) established the International Regulatory Co-operation for Herbal Medicines (IRCH), as part of a WHO Traditional Medicine Strategy [1]. Ensuring the efficacy, quality, and safety of herbal medicines is a key component of the strategy. The articles presented in this Special Issue address this component and represent a growing body of work in which a plethora of bioactive properties, of the therapeutic potential, of plants extracts have been identified. The obvious and consistent questions that arise from such studies are what are the contributions of the plants’ constituents to these properties and how significant is the therapeutic potential identified? The factors, which some might call challenges, that need to be considered in answering such a question effectively include the impact of different extraction techniques on the bioactive properties of plant extracts and developing and utilizing methods that simulate and model as closely as possible disease processes in humans. The aim of this Special Issue was to invite submissions that explored and addressed these factors as part of the process of gaining greater insights into how the bioactive properties of plant extracts (1) are conferred by their constituents and (2) can be exploited in developing new therapies for certain conditions.

## 2. Contributions

The studies that make up this Special Issue investigated extracts of, or compounds isolated from, plants, used in traditional medicine, indigenous to Africa, Asia, and South America as well as plants found worldwide. The properties identified and/or investigated include antidiarrheal, anti-inflammatory, anti-microbial, antioxidant, anti-mutagenic, and neuroprotective activity, with a number of phytochemicals, including phenolic acids, flavonoids, and terpenoids identified as contributors to some of these properties. Some of the studies investigated the impact of different extraction methods, including more advanced techniques [2,3], on constituent composition and/or biological activity: Wei and Zhang [2] demonstrated the effective use of supercritical fluid extraction with CO_2_ to prepare volatile oils of the leaf and stem of *Farfugiam japonicum*. They were able to identify and quantify 47 and 40 compounds from leaf and stem oils, respectively. On the basis of this analysis, they were able to attribute the enhanced anti-oxidizing and anti-bacterial activity of stem oil, compared with leaf oil, to higher proportions of benzenes, phenolics, and monoterpenes. The study by Gonzalez et al. [3] explored the possibility of optimizing extraction conditions using ultrasound-assisted extraction (UAE) and microwave-assisted extraction (MAE) on *Fabiana purensis*, with the prediction of optimized extraction conditions for UAE and MAE made possible using response surface methodology. They reported that compared to extracts obtained using maceration, HPLC-DAD patterns were similar but the extraction rates for the optimized extracts were faster (based on extraction yields). In addition, although all extracts exhibited comparable antioxidant, anti-inflammatory, and anti-mutagenic activities, the UAE had the strongest anti-inflammatory and anti-mutagenic activities. This study demonstrates the importance of developing techniques that optimize the extraction of bioactive compounds. Wojciak et al. [4] in their study on the antioxidant and anti-inflammatory effect of extracts of stinging nettle, *Urtica diocia*, reported that both an ethanol water extract (UdE) and a polyphenolic fraction (UdF) were rich in the phenolic acid caffeic acid and its derivatives, and via this composition, a protective effect was exhibited against oxidative stress and reactive oxygen species scavenging activity in normal human colon epithelial cells. The more potent of these activities was possessed by UdF and attributed to its greater concentration of polyphenols. Furthermore, this extract also showed some cytotoxic activity (albeit moderate) against human colorectal adenocarcinoma cells. Although not focused on different extraction methods, Ekaghba et al. [5] reported that harvesting season and age have an impact on the phytochemical levels of extracts of *Pentadesma butyracea* stem barks. For example, the phenolic contents of stem barks of the tree when harvested during the dry season in Gabon were higher than those obtained from barks harvested during the rainy season. The study by Carvalho et al. [6] on a polyphenolic-rich sugar cane straw extract, which investigated its anti-microbial properties for the purpose of prolonging the shelf life of cosmetics formulations, also demonstrated the importance of the extraction methods, specifically the use of different solubilizing agents on said activity.

In addition to their use in assessing the safety of the plant extracts/constituent investigated, animal and cell-based models were used to gain insights into the therapeutic potential of the properties of the plant investigated and revealed the complexity of the purported mechanisms of action of their extracts/constituents. Using primary cortical neurons, Liao et al. [7] reported that tetrahydroalstonine (THA) isolated from *Alstonia scholaris* protected against neuronal injury via autophagy regulation. This finding suggests that THA may possess neuroprotective properties that could be exploited for use in the treatment of ischemic stroke. The authors articulate the complexity of the regulation of cellular autophagy and acknowledge that to more fully understand the therapeutic potential of THA, work is required to identify its specific target in the treatment of this condition. El-Newary et al. [8] used an oral ulcer candidiasis model in immunosuppressed rats, which was created in three stages: immunosuppression, infection with Candida albicans, and treatment with juice obtained from fresh leaves of *Chenopodium murale*, a plant traditionally used to treat oral ulcers in newborn children in Egypt. The authors reported that the juice exhibited anti-fungal activity by the promotion of phagocytosis involving an increase in reactive oxygen species production as well as via immunomodulatory effects, which involved T cell activation and the subsequent production of cytokines, specifically IFN-gamma, IL-2, and IL-17. These activities were primarily attributed to the juice’s polyphenolic constitutes, including kaempferol and kaempferol-bound glycosides, gallic acid and chlorogenic acid, quercetin and naringenin, and its alkaloid constituents. In the study by Wójciak et al. [4], only the UdF extract exhibited moderate cytotoxic effects on the colon cancer cell line, and alongside this action, it also decreased the release of the pro-inflammatory cytokines IL-1β but not IL-6, suggesting that this extract might exert some therapeutic potential via its anti-inflammatory action. However, the authors acknowledge that more work is needed for the purposes of clarity regarding the extract’s therapeutic potential. As stem extracts of *Pentadesma butyracea* are traditionally used to treat diarrhea in Gabon, Ekaghba et al. [5] used in vitro (contractile activity of rat ileal smooth muscle) and in vivo (castor oil-induced diarrhea and enteropooling) models to demonstrate the extract’s ability to exhibit a relaxing effect on ileal smooth muscle and antidiarrheal activity, respectively, in a dose-dependent manner. The possible constituents that contributed to the activity in vitro were identified as polyphenols, mainly flavanone–flavone bioflavonoids. Furthermore, the in vivo model, specifically the mechanism by which castor oil induces diarrhea, provided possible insights into the antidiarrheal activity of the stem barks, including the inhibition of cyclooxygenase, resulting in a decrease in prostaglandin production or the activation of sodium/potassium ATPase channels. Both approaches provided greater understanding of the therapeutic potential of the stem extract and the amounts at which it is effective.

In summary, with a focus on particular plants, this Special Issue includes studies which provide further knowledge of the impact of different extraction techniques on the bioactive properties of plant extracts. The contribution of phytochemicals to these bioactive properties was also explored, as were the therapeutic potential and the mechanisms of action of some of the plant extracts/constituents of interest using in vitro and in vivo models. In different ways, this body of work contributes to providing further insights into how the bioactive properties of plant extracts are conferred by their constituents, and how their therapeutic potential can be exploited for the purposes of developing new therapies for certain conditions.
